# Reconstructed Three-Dimensional vs. Two-Dimensional Visualization in Unilateral Biportal Endoscopic Spine Surgery: A Randomized Crossover Simulation Feasibility Study

**DOI:** 10.3390/neurosci7050097

**Published:** 2026-08-31

**Authors:** Felix Corr, Silvio A. Heinig, Simon Behringer, Felix C. Stengel, Lorenzo Bertulli, Nader Hejrati, Oliver Bozinov, Martin N. Stienen, Stefan Motov

**Affiliations:** 1 Department of Neurosurgery & Interdisciplinary Spine Center, HOCH Health Ostschweiz, Cantonal Hospital of St. Gallen, 9007 St. Gallen, Switzerland; 2 Department of Neurosurgery, Medical Center, Faculty of Medicine, University of Freiburg, 79108 Freiburg, Germany

**Keywords:** depth perception, endoscopy, education, medical, lumbar vertebrae, simulation training, imaging, three-dimensional

## Abstract

Background/Objectives: Reconstructed three-dimensional (3D) endoscopic visualization is believed to improve depth perception and surgical performance compared with conventional two-dimensional (2D) systems. However, evidence supporting its use in unilateral biportal endoscopic (UBE) spine surgery remains limited. The objective was to compare procedural performance, task completion times, and subjective workload between 3D and 2D visualization in simulated biportal lumbar endoscopy. Methods: This randomized crossover pilot feasibility ex vivo study enrolled 6 neurosurgeons (3 residents, 3 seniors) who each completed a standardized UBE lumbar discectomy simulation (UpSurgeOn Lumbar SpineBox) under both 3D and 2D visualization in randomized sequence. The primary outcome was composite procedural performance (0–100 scale) assessed using a multi-item checklist encompassing technical success, quality, and safety. Secondary outcomes included task completion times for 7 procedural steps and subjective workload. Linear mixed-effects models adjusted for expertise level, spinal segment, operative side, and session order. Within-participant effect sizes (Cohen’s d) and a sensitivity power analysis were calculated. Results: All participants completed both conditions (24 procedures). Composite performance was high with both modalities, with no significant difference for the unweighted (adjusted difference 0.64 points, 95% CI −3.67 to 4.96, *p* = 0.76, d = 0.13) or weighted score (1.67 points, 95% CI −4.04 to 7.37, *p* = 0.55, d = 0.26). Subjective workload also did not differ (*p* = 0.67, d = 0.19). Effect sizes were negligible to small for most outcomes but reached medium magnitude for subjective fatigue (d = 0.54) and situational stress (d = 0.56). Exploratory expertise-stratified comparisons suggested faster lamina drilling with 3D among seniors (*p* = 0.04) and slower dura identification with 3D among residents (*p* = 0.02), based on only 3 participants per subgroup. The sample afforded 80% power to detect only very large effects (d ≈ 1.43). Conclusions: 3D visualization was feasible and well tolerated but did not significantly improve procedural performance, task completion time, or subjective workload in UBE simulation. Because the study was powered to detect only very large effects, these nonsignificant results reflect limited power rather than equivalence, and the medium effect-size signals for fatigue and situational stress are hypothesis-generating. Adequately powered trials are needed to confirm task-specific or expertise-dependent benefits.

## 1. Introduction

Unilateral biportal endoscopic (UBE) spine surgery, first described as translaminar lumbar epidural endoscopy in 1996 and formally introduced under the modern “biportal” designation in 2016, uses two independent ipsilateral portals to perform decompression, discectomy, and interbody fusion through a familiar interlaminar anatomic plane, combining the wide triangulated working angle of open microsurgery with the magnified, illuminated visualization of endoscopy [1,2,3]. Across the past decade indications have expanded from lumbar disc herniation and central/lateral recess stenosis to interbody fusion, posterior cervical foraminotomy, thoracic decompression, and revision surgery [4,5,6]. Prospective randomized controlled trials together demonstrate clinical equivalence to microscopic or anterior cervical fusion surgery with significantly less early surgical-site pain, lower serum creatine phosphokinase, and fewer wound complications [7,8,9,10,11]. A meta-analysis of 1001 patients [12] and the most recent pooled analysis [13] corroborate shorter hospital stay, lower estimated blood loss, and improved disability outcomes for UBE over open microdiscectomy, with equivalent leg-pain relief and complication profile. Pooled complication rates for UBE remain in the 5–8% range, dominated by incidental durotomy (2.5–4.5%) [10,14,15]. However, widespread adoption remains limited by a pronounced learning curve, with proficiency requiring substantial case volume, particularly because UBE demands coordinated bimanual instrument handling, continuous endoscopic orientation, and precise spatial judgment within a constrained operative corridor [16,17].

Conventional preoperative planning in spine surgery relies on mental integration of two-dimensional (2D) cross-sectional imaging into three-dimensional (3D) anatomical models, a cognitively demanding task that is particularly challenging for complex access trajectories and surgeons early in their learning curve [18]. Additionally, 3D virtual planning and volumetric reconstruction tools have been shown to improve trajectory accuracy, reduce intraoperative fluoroscopy exposure, and optimize access point planning in endoscopic lumbar procedures, with measurable benefits for procedural safety and efficiency [18,19]. Beyond preoperative applications, intraoperative 3D spatial orientation represents a critical performance determinant specific to the endoscopic corridor, where confined anatomy, continuous irrigation, and the absence of direct tissue feedback impose exceptional demands on depth perception and hand–eye coordination.

A recognized technical limitation of conventional 2D endoscopic systems is the absence of accurate binocular depth perception, which can impair spatial orientation and hand–eye coordination and may contribute to technical difficulties [20,21,22]. In non-spinal endoscopic disciplines, 3D visualization has been associated with improved technical performance, including 45% shorter task completion times for complex bimanual tasks, more than threefold lower error rates during suturing and rope-passing tasks, and overall reductions in performance time and error rates in 71% and 63% of randomized comparisons with 2D systems, respectively, with benefits observed across experience levels [23,24].

In endoscopic spine surgery, evidence supporting 3D visualization remains limited to early clinical series and pilot studies reporting improved depth perception and anatomical recognition, without controlled comparisons or sufficient power to evaluate performance or safety outcomes [21,25]. Moreover, no prior studies have examined the effects of 3D versus 2D visualization in standardized UBE simulation environments, where confounding by case complexity and surgeon experience can be minimized.

Given the pronounced learning curve associated with UBE [17] and the technical demands specific to this approach, defining the potential educational value of 3D visualization in a supervised simulation environment is clinically relevant. By potentially improving depth perception and spatial orientation, stereoscopic visualization may facilitate more efficient skill acquisition and smoother progression along the learning curve.

Accordingly, this pilot study was designed to estimate the direction and magnitude of differences in (1) composite procedural performance score and (2) step-specific task completion times between reconstructed 3D and 2D endoscopic visualization modalities during standardized UBE simulation, and to assess whether these effects differed by surgeon expertise level. These preliminary estimates were intended to inform outcome selection, sample-size planning, and the design of adequately powered future comparative studies.

## 2. Materials and Methods

This study was conducted in accordance with the Declaration of Helsinki, approved by the regional ethics committee (BASE-ID Req-2025-00973, EKOS 25/123), and prospectively registered on ClinicalTrials.gov (NCT07171801), with first participant enrollment on 6 October 2025. The study is reported in accordance with the CONSORT 2025 guidelines (Supplementary Table S1).

### 2.1. Trial Design & Setting

This randomized, blinded, crossover, pilot ex vivo feasibility trial used a 1:1 allocation ratio. Participants completed identical simulation tasks for UBE decompression and discectomy under reconstructed 3D and 2D visualization in randomized order, serving as their own controls. Randomization of modality sequence was used to mitigate learning and period effects. The study followed an exploratory estimation framework, designed to generate preliminary effect size estimates and assess feasibility. Given the feasibility and estimation objectives and the absence of reliable prior data for clinically meaningful within-participant differences in this standardized UBE simulation setting, no formal sample size calculation was undertaken.

**Figure 1:**
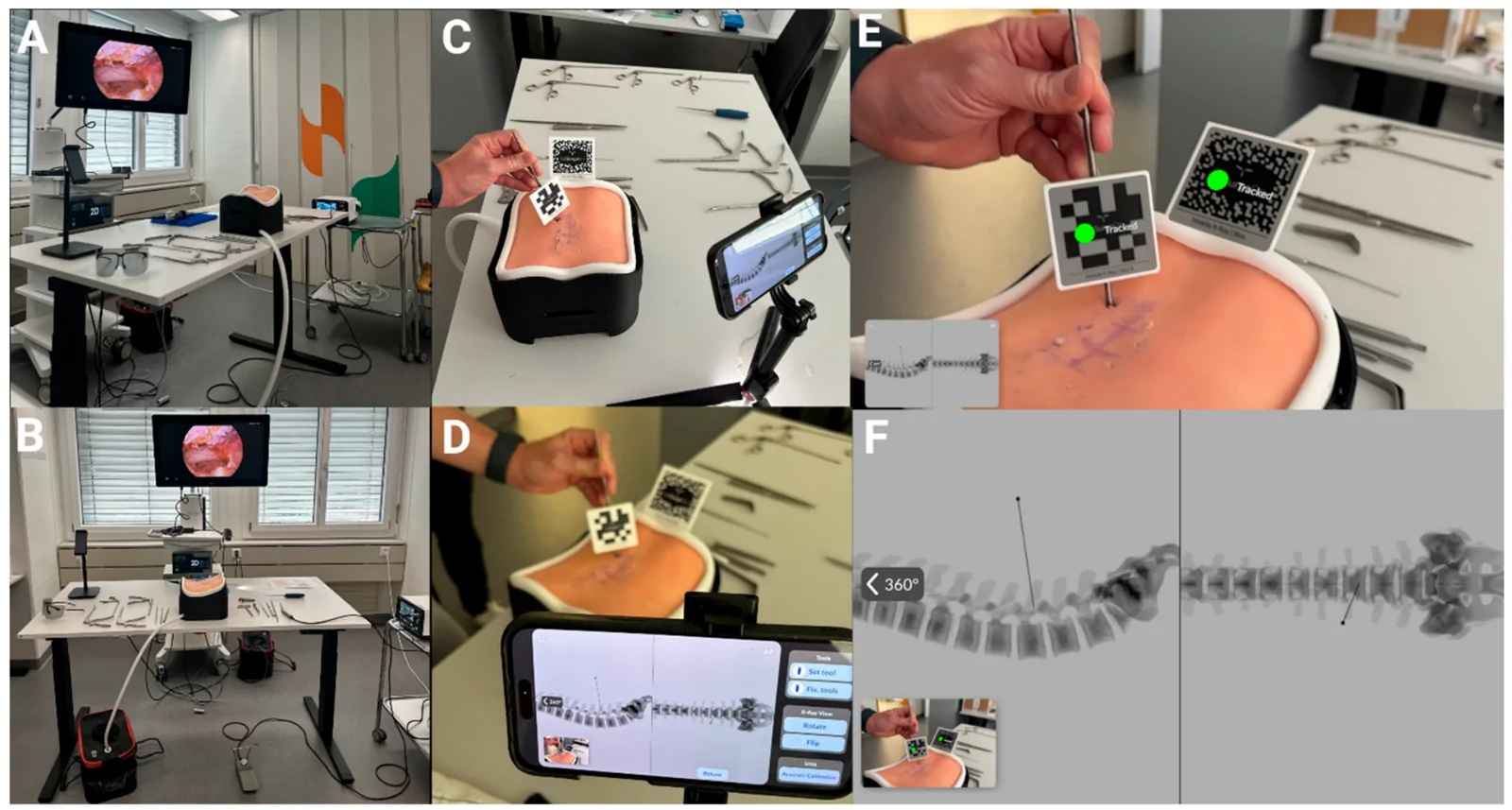
Simulation setup and augmented reality-assisted model registration. (**A**,**B**) Standardized endoscopic simulation workstation with endoscopic tower, instruments, and lumbar spine model. (**C**) Augmented reality (AR) tracking system using fiducial markers positioned on the lumbar spine model for spatial registration. (**D**) Mobile device interface displaying real-time AR visualization with fiducial marker detection overlay during model registration. (**E**) Fiducial marker placement on an anatomical model with dual circular markers (green dots indicate successful detection) for precise vertebral localization. (**F**) Virtual fluoroscopy view generated from AR registration, showing the lumbar spine in sagittal (**left**) and axial (**right**) orientations, enabling digital anatomical navigation and verification of model alignment before access-point planning. No intraoperative navigation or real-time guidance was provided during simulation task performance.

The study was conducted in the neurosurgical training room of the Department of Neurosurgery, HOCH Health Ostschweiz, Cantonal Hospital of St. Gallen, Switzerland, a purpose-built facility for advanced microsurgical and endoscopic training. All sessions were performed at a standardized ergonomic workstation with a validated spinal simulation model and identical instrumentation, under uniform lighting conditions (Figure 1A,B).

### 2.2. Eligibility Criteria

Eligible participants were neurosurgical residents and board-certified senior neurosurgeons at the Department of Neurosurgery at HOCH Health Ostschweiz, Cantonal Hospital of St. Gallen, Switzerland. Inclusion criteria were participation in the institutional training program and prior basic theoretical knowledge concerning UBE. Exclusion criteria included substantial prior reconstructed 3D endoscopy experience (>10 independent procedures), conditions affecting stereopsis or fine motor control, and inability to complete the simulation protocol. Participants were recruited by voluntary departmental invitation without incentives.

### 2.3. Intervention and Comparator

Each participant completed standardized spinal endoscopic simulation tasks for UBE unilateral decompression and discectomy under reconstructed 3D (intervention) and 2D (comparator) visualization, with stereoscopic activation of the endoscopy screen as the sole experimental difference. In the reconstructed 3D condition, stereopsis was enabled via a stereoscopic image-processing system (MonoStereo MS-301, MedicalTek Co., Ltd., Taichung City, Taiwan), which converted conventional 2D endoscopic input into real-time stereoscopic output; in the 2D condition, stereoscopy was disabled. Accordingly, the investigated system represents reconstructed rather than native dual-optical-channel 3D visualization. Endoscopic visualization was provided using a 4K ultra-high-definition camera system coupled to a 0° rigid spinal endoscope (Karl Storz SE & Co. KG, Tuttlingen, Germany) and displayed on a medical-grade 3D-capable monitor (Barco MDSC-8232 M3D, Barco, Kortrijk, Belgium). Camera input resolution was 3840 × 2160 at 60 Hz, with stereoscopic output displayed at 1920 × 1080. Display parameters, including brightness, contrast, and gamma, were standardized and remained unchanged across visualization modes. Identical disposable passive-polarized 3D eye shields (CFV-B100, Sony, Tokyo, Japan) were used for all participants and replaced between sessions. Participants were monitored for visual discomfort or other 3D-related adverse effects. Continuous irrigation was delivered using a pressure-controlled fluid management system (Stöckli Medical AG, Bern, Switzerland) at a standardized pressure of 35 mmHg and flow rate of 1.5 L/min. Participants completed an identical predefined sequence of validated tasks during two separate sessions separated by a standardized rest interval of 24 h, with visualization order randomized. Standardized instructions and faculty supervision ensured procedural fidelity, and no adjunct technologies were permitted. Potential order and carryover effects were addressed by randomizing sequence allocation and adjusting for session order in the statistical analyses.

### 2.4. Simulation Task and Procedural Protocol

**Figure 2:**
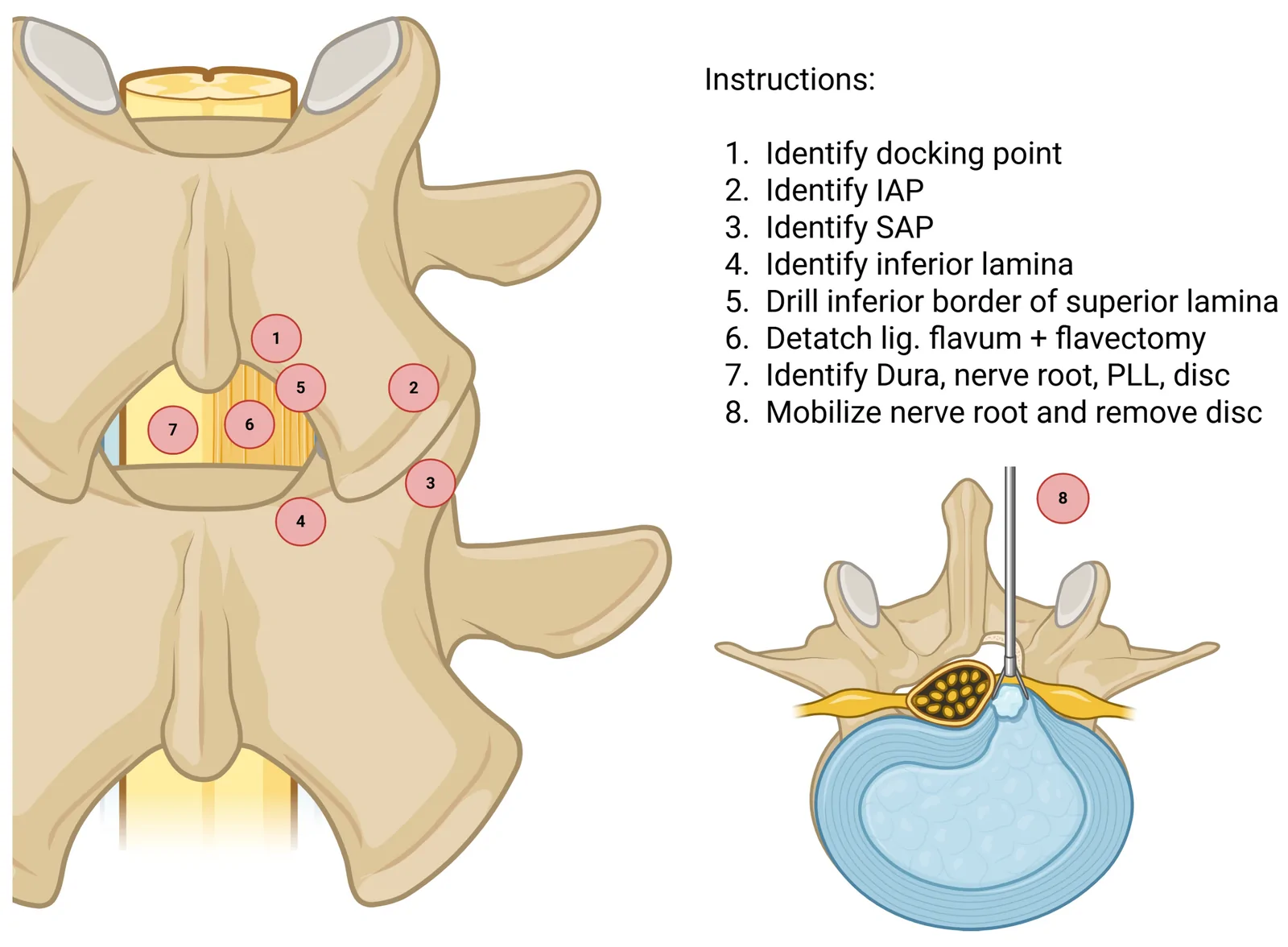
Procedural workflow and task sequence for standardized unilateral biportal endoscopic lumbar discectomy simulation. Posterior (**left**) and axial (**right**) anatomical views of the lumbar spine illustrating the eight prespecified procedural steps. Numbered markers indicate the approximate anatomical target for each step (created with Biorender.com). Abbreviations: IAP, inferior articular process; PLL, posterior longitudinal ligament; SAP, superior articular process.

Before task initiation, the simulation model was registered using augmented reality-based tracking to standardize biportal access points (Figure 1C–F). Participants then performed a standardized single-level biportal unilateral endoscopic decompression and discectomy on an anatomically validated lumbar spine model (UpSurgeOn Endoscopic LumbarBox; Upsurgeon©, Milan, Italy) following a prespecified procedural sequence reflecting routine clinical practice (Figure 2).

Spinal level and operative side were determined by the availability of previously unused segments within pre-used simulation models. Accordingly, only unused levels and sides were utilized to avoid repeated manipulation of the same anatomical region. All participants received identical written instructions. Bony decompression was performed using a high-speed drill (Primado2^®^, VOSTRA GmbH, Aachen, Germany), and a standard endoscopic instrument tray (BONSS, Nexon Medical AG, Oberkirch, Switzerland) was used. No navigation, fluoroscopy, or adjunct technologies were permitted. The simulation model, instrumentation, task sequence, and operating conditions were identical across sessions, isolating visualization modality as the sole experimental variable.

### 2.5. Outcomes

The primary outcome was procedural performance, assessed using a prespecified standardized checklist covering anatomical identification, bone work, nerve root and disc management, and safety-related events (Supplementary Material Table S2).

The checklist was developed de novo for this study, as no previously validated assessment tool exists for standardized UBE simulation performance. The checklist content was developed through expert consensus among the faculty neurosurgeons involved in UBE training prior to study initiation. The checklist comprised 17 items across three categories: nine binary success items (scored 0–1), capturing discrete task completion endpoints; three ordinal quality items (scored 0–2), capturing graded performance quality for anatomical identification, bone work, and nerve root mobilization; and five adverse event items (binary, reverse-coded: event present = 0, no event = 1), capturing safety-relevant outcomes including dural tear, nerve root trauma, and instrument misdirection. All items were direction-coded so that higher scores reflected better performance.

The unweighted composite score was calculated as the sum of all item scores divided by the maximum achievable raw sum, multiplied by 100 (0–100 scale), treating all items as equally important. The weighted composite score assigned differential weights a priori based on clinical reasoning, binary success items (weight 1), ordinal quality items (weight 2), and adverse event items (weight 3), such that avoidance of a safety-relevant event contributed three times as many points to the composite score as completion of a binary task endpoint. The weighted raw sum was normalized identically to a 0–100 scale. Both scores are reported to allow assessment of result robustness independent of the weighting scheme. Formal psychometric validation was not performed, as the checklist was designed to capture procedurally distinct performance domains rather than a unidimensional construct. Internal consistency metrics are therefore not applicable to this instrument type.

Procedural performance was assessed in real time by two faculty neurosurgeons who were both present for all simulation sessions. Scores were determined by consensus following joint discussion, and no procedure was rated by a single assessor independently. Raters underwent calibration before study initiation. Task completion time for each of the seven predefined procedural steps was recorded in seconds from endoscope insertion to successful task completion.

Secondary outcomes included subjective workload assessed using the NASA Task Load Index (NASA-TLX), a validated multidimensional instrument measuring mental demand, physical demand, temporal demand, effort, performance, and frustration, yielding a composite score from 0 (lowest workload) to 100 (highest workload) [26]. For each session, a raw NASA-TLX score was calculated as the unweighted mean of the six subscale ratings. Additional secondary outcomes included user experience ratings (0–100 visual analog scales), ergonomic assessments (5-point Likert scales), and binary ratings of visual quality, as previously described [27]. All secondary outcomes were recorded immediately after each simulation session.

### 2.6. Sequence Generation & Blinding

The order in which participants completed the two visualization conditions was randomized so that each participant performed both 2D and 3D simulations. The allocation sequence was generated by an independent investigator using a computerized random number generator (R software, version 4.2.1) with a 1:1 ratio, and the allocation was implemented using sequentially numbered, opaque, sealed envelopes opened immediately before task initiation. Participants and real-time outcome assessors were not blinded because the visualization modality is inherently perceptible. To mitigate bias, outcomes were prespecified using objective criteria, data were coded with anonymized condition labels.

### 2.7. Statistical Methods

All analyses were performed in R version 4.4.0 (R Foundation for Statistical Computing). Continuous variables are presented as mean (SD), ordinal variables as median [interquartile range], and categorical variables as number (percentage). Group comparisons of categorical variables were performed using Fisher’s exact tests, as appropriate. Composite performance scores were analyzed using linear mixed-effects models with visualization modality, expertise level, and their interaction as fixed effects, adjusting for spinal segment, operative side, and session number, with participant as a random intercept. Estimated marginal means were computed by expertise level, and within-participant differences were assessed using paired *t*-tests. Secondary outcomes, including task completion times, subjective workload (NASA Task Load Index), and user experience measures, were analyzed using linear mixed-effects models, cumulative link mixed models (ordinal outcomes), or mixed-effects logistic regression (binary outcomes), with the same covariate structure. Binary outcomes with complete separation are reported descriptively. All mixed-effects models used restricted maximum likelihood estimation with Kenward-Roger degrees-of-freedom approximation. Within-participant effect sizes (Cohen’s d, calculated from the standard deviation of the within-participant differences) were calculated for all continuous outcomes. Because this was a pilot trial not powered for hypothesis testing, a sensitivity analysis was used to define the detectable effect: 6 participants afforded 80% power (2-sided α = 0.05) to detect only a very large paired effect (d ≈ 1.43), and effect sizes are reported as point estimates to inform future sample-size estimation rather than to support inferential claims. A two-sided *p*  ≤  0.05 was considered statistically significant. No adjustments for multiple comparisons were applied due to the pilot design. No missing data was observed.

## 3. Results

### 3.1. Participant Flow

Six right-handed neurosurgeons (three residents, three seniors) completed 24 simulation procedures. Each participant performed four sessions (two with 2D, two with 3D visualization) in a randomized sequence, with four participants (66.7%) starting with 2D and two (33.3%) starting with 3D visualization. Simulations spanned five lumbar levels, most commonly L2/L3 (*n* = 8, 33.3%) and L5/S1 (*n* = 7, 29.2%), with a left-sided approach in 14 procedures (58.3%) and a right-sided approach in 10 (41.7%) (Table 1). Distribution of visualization modality, spinal level, operative side, and randomization sequence did not differ significantly between residents and seniors. There were no exclusions, protocol deviations, or losses to follow-up.

### 3.2. Composite Performance Score

**Figure 3:**
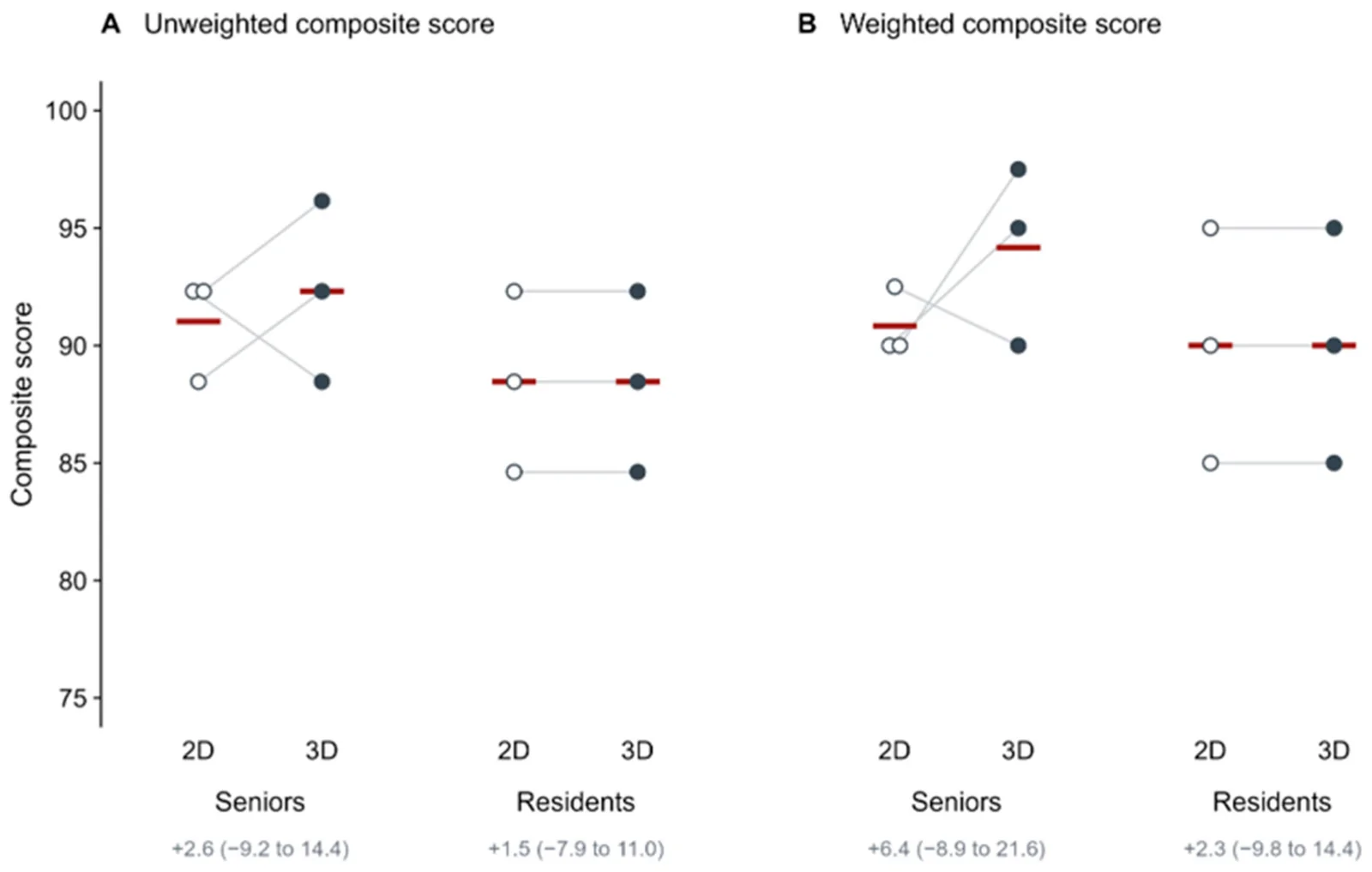
Composite performance under 2D and 3D endoscopic visualization, shown separately for senior surgeons and residents (*n* = 3 each). (**A**) Unweighted composite score. (**B**) Weighted composite score. Open circles indicate 2D and filled circles 3D visualization. Each circle represents one participant, averaged across the two procedures performed under the respective modality, and grey lines connect the paired observations of the same participant. Participants with identical scores are displaced horizontally so that all individual values remain visible, and the horizontal position carries no further meaning. Red bars indicate group means. Values below each group give the adjusted mean difference (3D minus 2D) with its 95% confidence interval, derived from the mixed-effects models reported in Table 2, with positive values favouring 3D visualization.

Overall composite performance was high with both visualization modalities (Table 2 and Figure 3). The mean unweighted composite score was 89.7 ± 5.0 with 2D visualization and 90.4 ± 5.8 with 3D visualization, while the weighted composite score was 90.4 ± 6.6 with 2D and 92.1 ± 6.9 with 3D. Performance was similarly high across experience levels. Among seniors, unweighted scores were 91.0 ± 3.1 with 2D and 92.3 ± 4.9 with 3D, and weighted scores were 90.8 ± 4.9 and 94.2 ± 4.9, respectively. Among residents, unweighted scores were identical between modalities (88.5 ± 6.4 for both), as were weighted scores (90.0 ± 8.4 for both). Item-level success rates for anatomical identification, decompression steps, and adverse event avoidance were uniformly high in both conditions (≥91.7%). The only notable task-level difference was in Kerrison flavectomy technique, where en bloc removal was achieved in 16.7% of 2D procedures compared with 8.3% of 3D procedures.

Adjusted mixed-effects models showed no statistically significant difference between 3D and 2D visualization for either composite score (all *p* > 0.05), consistent with the limited statistical power of this pilot sample (unweighted: adjusted difference, 0.64 points favoring 3D; 95% CI, −3.67 to 4.96; weighted: 1.67 points; 95% CI, −4.04 to 7.37). Subgroup and interaction analyses were likewise nonsignificant (modality × expertise: unweighted, *p* = 0.87; weighted, *p* = 0.64). Nevertheless, effect-size estimation revealed negligible-to-small within-participant effects overall (unweighted d = 0.13; weighted d = 0.26), with a medium point estimate for the weighted score among seniors (d = 0.69; vs. d ≈ 0.01 among residents). As all confidence intervals crossed zero and all effects fell below the d ≈ 1.43 detectable in this sample, these estimates are reported as imprecise, hypothesis-generating signals rather than evidence of a true difference or of equivalence between modalities.

### 3.3. Task Completion Time

No statistically significant differences in task completion time were observed between 3D and 2D visualization for any procedural step in pooled analysis (all *p* > 0.05, Table 3), consistent with the limited power of this pilot sample. Within-participant effect sizes were nonetheless non-trivial for several steps. Small-to-medium estimates favored 3D for dura identification (d = 0.58), nerve root mobilization (d = 0.49), and posterior longitudinal ligament opening (d = 0.41), and a small estimate favored 2D for lamina drilling (d = 0.39). All confidence intervals crossed zero and fell below the d ≈ 1.43 detectable in this sample. Two expertise-stratified comparisons reached nominal significance. Lamina drilling was faster with 3D among seniors (mean difference, −101 s, 95% CI, −195 to −6, *p* = 0.04) and dura identification was slower with 3D among residents (563 s, 95% CI, 102 to 1024, *p* = 0.02). However, these subgroups comprised three participants each, were not corrected for multiple comparisons, and carried wide confidence intervals. They are therefore reported as exploratory, hypothesis-generating observations only and should not be interpreted as reliable modality effects.

### 3.4. Subjective Workload

**Figure 4:**
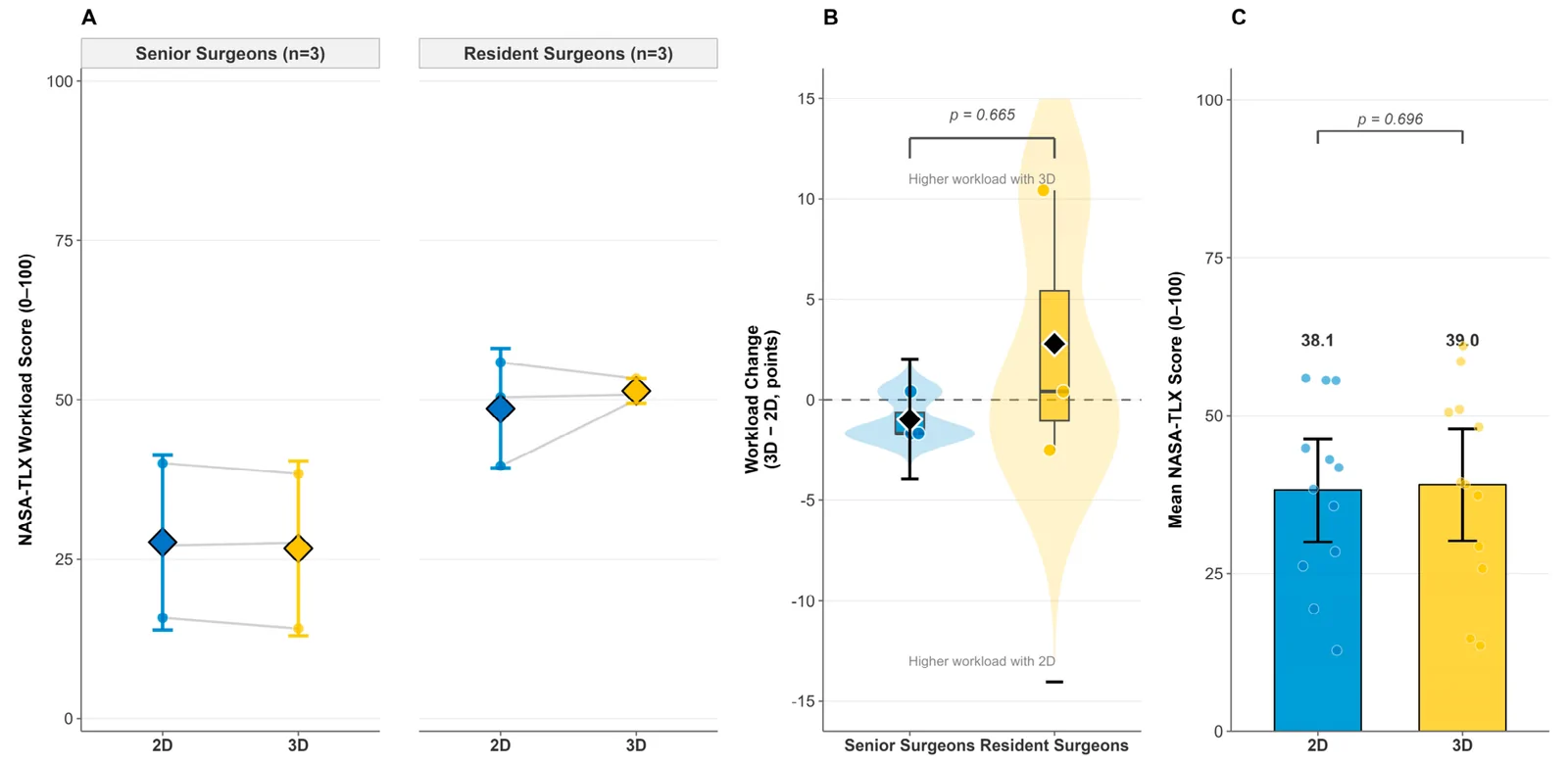
Comparison of subjective workload between 2D (blue) and 3D (yellow) endoscopic visualization using the NASA-TLX. (**A**) Individual participant scores (diamonds represent means, error bars show standard deviations) for senior surgeons (*n* = 3, (**left panel**)) and resident surgeons (*n* = 3, (**right panel**)), with connecting lines indicating within-participant changes between visualization modalities. (**B**) Violin plot illustrating the distribution of within-participant workload changes (3D minus 2D), with box plots showing median and interquartile ranges. Positive values indicate a higher workload with 3D visualization. No significant difference was observed between modalities (*p* = 0.665). (**C**) Overall mean NASA-TLX scores by visualization modality. Bar graphs show adjusted estimated marginal means with 95% confidence intervals; individual data points represent participant scores.

No significant difference in workload was observed between 3D and 2D visualization (adjusted mean difference, −1.6 points, 95% CI, −9.7 to 6.5, *p* = 0.67, Table 4, Figure 4), consistent with the limited power of this pilot sample. Among seniors, adjusted mean scores were 26.8 (95% CI, 11.8 to 41.8) with 2D versus 25.2 (95% CI, 10.1 to 40.2) with 3D, a difference of 1.6 points (95% CI, −6.6 to 9.8, *p* = 0.67). Among residents, adjusted mean scores were 47.1 (95% CI, 32.1 to 62.1) with 2D versus 50.6 (95% CI, 35.6 to 65.6) with 3D, a difference of −3.5 points (95% CI, −12.8 to 5.8, *p* = 0.42). The interaction between modality and expertise level was not significant (*p* = 0.35). In pooled analysis collapsing across expertise levels, the overall effect remained nonsignificant (mean difference, −0.9 points, 95% CI, −5.7 to 3.9, *p* = 0.70). The overall within-participant effect size was negligible (d = 0.19). Larger point estimates within the expertise subgroups (seniors d = 0.81, residents d = 0.41) were based on three participants each, were highly imprecise, and fell well below the d ≈ 1.43 detectable in this sample. They are reported for completeness only and should not be interpreted as modality effects. Residents reported a higher overall workload than seniors irrespective of modality, a difference that approached but did not reach significance (adjusted mean difference, 20.3 points, 95% CI, −1.1 to 41.8, *p* = 0.06).

### 3.5. User Experience and Ergonomics

User experience and ergonomic outcomes were similar between 2D and 3D visualization (Table 5). Mean satisfaction with performance was 61.8 ± 26.7 with 2D and 67.2 ± 22.7 with 3D (adjusted mean difference, 2.0 points, 95% CI, −39.0 to 43.1, d = 0.14). No statistically significant differences were found between modalities for fatigue, distraction, task complexity, or situational stress (all *p* > 0.05), which is likely due to the relatively small sample size of this pilot study. Nevertheless, effect-size calculation revealed a medium difference for fatigue (21.8 ± 20.9 with 2D versus 26.2 ± 23.0 with 3D, Cohen’s d = 0.54) and for situational stress (18.5 ± 20.2 versus 17.0 ± 17.5, Cohen’s d = 0.56), with a small difference for task complexity (39.7 ± 24.5 versus 43.2 ± 25.3, d = 0.37). However, these estimates were imprecise, with confidence intervals crossing zero, and should therefore be interpreted descriptively and as hypothesis-generating rather than as evidence of a between-modality difference. Ordinal ratings for surgical ergonomics and working zone adequacy were high and comparable between modalities (median, 4 on a 5-point scale). Binary image-quality assessments showed high overall satisfaction with both modalities, although depth of field was rated satisfactory in 100% of 2D versus 91.7% of 3D sessions, image sharpness in 100% versus 66.7%, and monitor adjustment was required more frequently with 3D (33.3% versus 8.3%).

**Figure 5:**
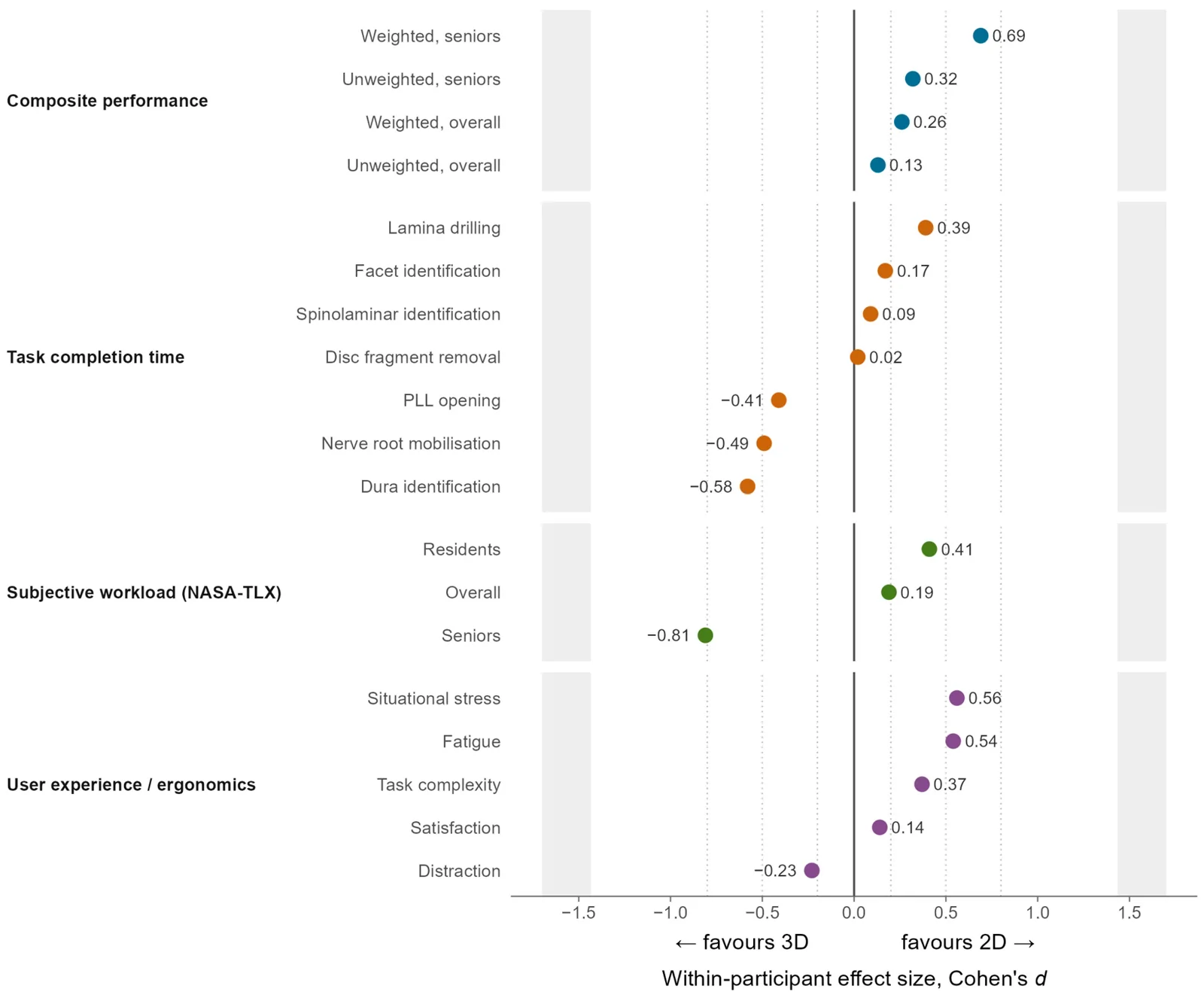
Within-participant effect sizes (Cohen’s d) for all continuous outcomes, grouped by domain, comparing 3D and 2D endoscopic visualization. Each point represents the standardized within-participant effect for one outcome, with the numeric value shown alongside. The solid vertical line marks no effect (d = 0). Dotted vertical lines mark the conventional benchmarks for small (0.2), medium (0.5), and large (0.8) effects. The shaded regions mark effects large enough to be detectable at 80% power given the achieved sample (|d| ≥ 1.43, *n* = 6); all observed effects fall within the unshaded central region, indicating that the study was underpowered to detect them. Negative values favour 3D visualization and positive values favour 2D visualization. Subgroup estimates for seniors and residents derive from 3 participants each and are correspondingly imprecise. Abbreviations: 2D, two-dimensional visualization; 3D, three-dimensional visualization; d, Cohen’s d (standardized within-participant effect size based on the standard deviation of the within-participant differences); NASA-TLX, National Aeronautics and Space Administration Task Load Index; PLL, posterior longitudinal ligament.

An overview of the within-participant effect sizes for all continuous outcomes, together with the effect detectable at 80% power, is shown in Figure 5.

## 4. Discussion

### 4.1. Educational and Training Implications

In this standardized simulation, 3D visualization was feasible but did not significantly improve composite procedural performance, task completion time, or subjective workload relative to 2D visualization. Effect sizes for the primary performance outcomes were negligible to small (d = 0.13 to 0.26), and the sample was powered to detect only very large effects (d ≈ 1.43), so these results indicate limited power rather than true equivalence. The most consistent between-modality difference was observed not in performance but in visual experience. Image sharpness, resolution, and luminance were rated lower with 3D, monitor adjustment was more often required, and subjective fatigue showed a medium effect-size increase (d = 0.54).

These findings align with a broader comparative literature that is methodologically heterogeneous and not uniformly supportive of 3D superiority. Although several studies report benefits of stereoscopic visualization for task efficiency and depth perception [28,29,30], a comparable body of evidence finds no significant difference in objective performance between modalities [30,31,32]. Many studies favoring 3D share the constraints of the present work, including small samples, inability to blind participants, and single-session assessment without longitudinal follow-up [33]. Reported advantages during demanding tasks among experienced users have been inconsistently replicated [33] and should not be assumed to generalize beyond their specific experimental contexts.

A similar pattern has been reported beyond simulation settings in clinical spine surgery. Early experience with stereoscopic endoscopic systems, including a series of 38 patients undergoing 3D biportal endoscopic surgery for lumbar degenerative disease, suggests improved depth perception and anatomical visualization but remains limited to small series and case reports without controlled performance comparisons [21,25]. The most extensive comparative evidence concerns the 3D exoscope, where systematic reviews and a procedure-specific meta-analysis against the operating microscope report comparable surgical and clinical outcomes, with advantages concentrated in ergonomics, maneuverability, and intraoperative teaching rather than in measurable performance [34]. Even within this literature the ergonomic effect is not uniform, as surgeon-reported maneuverability and fatigue remain points of disagreement [35], paralleling the medium fatigue signal observed with 3D in the present study. Three-dimensional technology is also being applied beyond the operative field, with 3D posturography used to quantify sagittal lumbosacral alignment and its postoperative and diurnal changes after lumbar discectomy, illustrating the widening role of 3D approaches across the spine-care pathway [36].

Importantly, performance scores were uniformly high across both modalities, including among residents without prior endoscopic experience, suggesting that simulation-based UBE training is feasible using either visualization system. Accordingly, the present findings do not support clear superiority of one modality for surgical training within this controlled experimental setting. Rather, selection of visualization technology may currently depend more on institutional resources, user preference, and platform availability than on established performance advantages. These observations complement previous work supporting structured simulation-based UBE training environments [37].

### 4.2. Performance Patterns by Expertise Level

Two expertise-stratified comparisons reached nominal significance. Among seniors, lamina drilling was faster with 3D (mean difference −101 s, *p* = 0.04), whereas among residents, dura identification was slower with 3D (563 s, *p* = 0.02). Both estimates derive from 3 participants per subgroup, were uncorrected for multiple comparisons, and carried wide confidence intervals, and therefore should be regarded as hypothesis-generating. Their fragility is illustrated by dura identification, for which the pooled within-participant effect favored 3D (d = 0.58) while the resident subgroup showed the opposite direction. Such direction reversal on subgroup splitting is characteristic of unstable estimates at this sample size and precludes any conclusion about an expertise-by-modality interaction, consistent with the nonsignificant interaction terms in the primary models.

Given the very small expertise subgroups (*n* = 3 each), these observations are too imprecise to support conclusions regarding expertise-dependent effects and may represent chance findings. They are therefore presented as exploratory observations. Whether stereoscopic visualization has task-specific or expertise-dependent effects requires confirmation in adequately powered randomized controlled clinical studies.

### 4.3. Feasibility and Technical Considerations

All participants completed both conditions, supporting the feasibility of the study protocol and the controlled crossover evaluation within a standardized UBE simulation environment. This should not be interpreted as demonstrating the clinical feasibility of reconstructed 3D visualization, which has already been described in early clinical reports. Rather, the simulation setting provided a controlled framework for within-participant comparison while minimizing clinical heterogeneity. Notable technical differences emerged. Participants reported reduced peripheral clarity and stereoscopic fidelity with 3D, particularly when monitor positioning deviated from a neutral viewing angle, and image sharpness, resolution, and luminance were rated lower than with 2D. These observations are consistent with the known limitations of passive-polarization displays, in which crosstalk and ghosting degrade stereoscopic perception under suboptimal viewing geometry [38,39] and may lead to more frequent monitor adjustment during 3D sessions. The medium effect-size increase in subjective fatigue with 3D (d = 0.54) and the medium-magnitude estimate for situational stress (d = 0.56) are directionally compatible with the increased perceptual demand of a degraded stereoscopic image, although neither reached significance and the situational-stress direction was inconsistent across raw and adjusted estimates. These outcomes may therefore warrant further evaluation, alongside objective image-quality metrics, in adequately powered future studies. However, the present data do not establish a between-modality difference. Overall NASA-TLX workload did not differ (d = 0.19), suggesting that any perceptual burden did not translate into a global increase in cognitive load within this short simulation, and residents reported higher workload than seniors irrespective of modality (*p* = 0.06), consistent with their concurrent acquisition of the UBE technique. NASA-TLX scores in both groups fell within ranges previously reported for simulation-based evaluations of advanced visualization and augmented reality systems [40,41].

### 4.4. Strengths and Limitations

Strengths of this study include the randomized crossover design, standardized simulation environment, complete outcome capture, and within-participant comparisons, which reduced interindividual variability. The use of anatomically realistic simulation models and prespecified objective performance metrics further enhanced methodological consistency.

Several limitations should be considered. First, this was a single-center pilot study of only six participants, resulting in limited statistical power and wide confidence intervals. Accordingly, the present study was not intended to provide definitive estimates of comparative effectiveness, and its findings should be regarded as exploratory and not generalized beyond the standardized simulation setting studied. A sensitivity analysis indicated that the sample afforded 80% power to detect only very large within-participant effects (d ≈ 1.43), which exceeds the magnitude of any effect observed. Accordingly, the absence of statistically significant differences should not be interpreted as evidence of equivalence between modalities, as the study was powered neither to detect nor to exclude clinically meaningful differences. The medium effect-size signals for subjective fatigue and situational stress are therefore best regarded as hypothesis-generating estimates that should inform the sample-size calculation of future confirmatory trials. Second, the simulation environment, if realistic high-fidelity models have been utilized, cannot fully reproduce important aspects of real UBE surgery, including bleeding, tissue variability, patient-specific anatomy, and intraoperative stress. Accordingly, the present findings should be interpreted as evidence regarding technical feasibility and comparative performance under standardized simulation conditions and should not be extrapolated to clinical effectiveness, patient outcomes, or intraoperative safety. Third, participants and assessors could not be blinded to visualization modality, introducing potential performance and expectancy bias inherent to visualization-based surgical training studies [42].

Furthermore, only a single commercially available 2D/3D platform was evaluated, limiting generalizability to other stereoscopic systems with different optical and ergonomic properties. The large number of subgroup and task-specific analyses relative to sample size increases the risk of false-positive findings and supports cautious interpretation of all exploratory subgroup observations, particularly the expertise-stratified comparisons based on 3 participants per group.

Additional methodological limitations relate to the crossover design. Randomization sequence was imbalanced across expertise groups, with all residents allocated to the 2D-first condition and most senior surgeons to the 3D-first condition, introducing the possibility of residual period and learning effects despite adjustment for session order. Similarly, the standardized 24-h interval between sessions may not have fully eliminated carryover effects given the identical procedural workflow. Finally, two outcome-assessment limitations warrant note. The primary outcome relied on a de novo composite checklist without formal psychometric validation. The checklist content was developed through expert consensus, and results were consistent across unweighted and weighted scoring. Nevertheless, findings based on the composite performance score should be interpreted as cautiously and require confirmation using a formally validated UBE-specific assessment instrument. In addition, the uniformly high composite performance scores in both visualization conditions raise the possibility of a ceiling effect. The assessment instrument may therefore have had limited discriminatory sensitivity for detecting subtle between-modality differences. Procedural performance was also scored by real-time consensus of two faculty assessors rather than by independent duplicate assessment, which reduced single-rater error but precludes a formal inter-rater reliability estimate and may have introduced observer-related bias. Independent duplicate scoring with reliability assessment should be incorporated into future confirmatory trials.

## 5. Conclusions

In this pilot ex vivo simulation study of unilateral biportal endoscopic spihne surgery, 3D visualization was feasible and well tolerated but was not associated with statistically significant improvements in procedural performance, task completion time, or subjective workload compared with 2D visualization. Because the sample provided 80% power to detect only very large effects (d ≈ 1.43), these nonsignificant results reflect limited power rather than equivalence, and the medium effect-size signals observed for subjective fatigue and situational stress should be interpreted as hypothesis-generating. Adequately powered trials are needed to determine whether 3D visualization offers task-specific or expertise-dependent benefits.

## Figures and Tables

**Table 1:** Procedural Characteristics by Expertise Level.

Characteristic	Overall, No. (%)	Residents, No. (%)	Seniors, No. (%)	*p*-Value
**Total procedures**	24	12	12	
**Visualization modality**				>0.99
2D	12 (50.0)	6 (50.0)	6 (50.0)	
3D	12 (50.0)	6 (50.0)	6 (50.0)	
**Spinal level**				0.49
L1/L2	3 (12.5)	2 (16.7)	1 (8.3)	
L2/L3	8 (33.3)	3 (25.0)	5 (41.7)	
L3/L4	2 (8.3)	0 (0.0)	2 (16.7)	
L4/L5	4 (16.7)	3 (25.0)	1 (8.3)	
L5/S1	7 (29.2)	4 (33.3)	3 (25.0)	
**Operative side**				>0.99
Left	14 (58.3)	7 (58.3)	7 (58.3)	
Right	10 (41.7)	5 (41.7)	5 (41.7)	
**Randomization sequence**				0.40
2D first	4 (66.7)	3 (100.0)	1 (33.3)	
3D first	2 (33.3)	0 (0.0)	2 (66.7)	

Abbreviations: 2D, two-dimensional; 3D, three-dimensional.

**Table 2:** Composite Performance Scores by Visualization Modality and Expertise Level.

Metric	Group	2D, Mean ± SD	3D, Mean ± SD	Adjusted EMM, 2D (95% CI) ^a^	Adjusted EMM, 3D (95% CI) ^a^	Difference (2D vs. 3D), Mean (95% CI)	*p* Value	Cohen’s d
	Senior	91.0 ± 3.1	92.3 ± 4.9	88.3 (80.0–96.7)	91.0 (84.0–97.9)	−2.6 (−14.4 to 9.2)	0.62	0.32
**Unweighted score (0–100)**	Resident	88.5 ± 6.4	88.5 ± 6.4	86.1 (79.2–93.0)	87.6 (77.8–97.5)	−1.5 (−11.0 to 7.9)	0.72	0.01
	**Overall**	89.7 ± 5.0	90.4 ± 5.8	89.7 (86.0–93.5) ^b^	90.4 (86.4–94.3) ^b^	−0.6 (−5.0 to 3.7) ^b^	0.76	0.13
	Senior	90.8 ± 4.9	94.2 ± 4.9	86.6 (76.1–97.2)	93.0 (84.3–101.7)	−6.4 (−21.6 to 8.9)	0.36	0.69
**Weighted score (0–100)**	Resident	90.0 ± 8.4	90.0 ± 8.4	86.3 (77.6–94.9)	88.5 (76.1–101.0)	−2.3 (−14.4 to 9.8)	0.68	0.01
	**Overall**	90.4 ± 6.6	92.1 ± 6.9	90.4 (86.2–94.6) ^b^	92.1 (87.8–96.3) ^b^	−1.7 (−7.4 to 4.0) ^b^	0.55	0.26

Abbreviations: CI, confidence interval; EMM, estimated marginal mean; SD, standard deviation. ^a^ Estimated marginal means from mixed-effects models adjusted for spinal segment, operative side, and session number using the Kenward–Roger degrees-of-freedom approximation. Negative differences favor 3D visualization (higher performance). ^b^ Pooled analysis collapsing across expertise levels.

**Table 3:** Task Completion Times by Procedural Step, Visualization Modality, and Expertise Level.

Task Step	Overall Effect (2D vs. 3D), Sec (95% CI) ^a^	*p* Value	Cohen’s d	Seniors (2D vs. 3D), Sec (95% CI)	*p* Value	s	*p* Value
Spinolaminar Junction identification	4 (−36 to 44)	0.84	0.09	−3 (−61 to 55)	0.92	65 (−2 to 132)	0.06
Facet joint identification	13 (−48 to 74)	0.66	0.17	−55 (−155 to 45)	0.25	73 (−42 to 188)	0.19
Lamina drilling	31 (−32 to 93)	0.31	0.39	−101 (−195 to −6)	0.04	51 (−57 to 158)	0.32
Dura identification	−201 (−486 to 85)	0.16	−0.58	201 (−199 to 601)	0.29	563 (102 to 1024)	0.02
Nerve root mobilization	−146 (−480 to 188)	0.37	−0.49	190 (−269 to 648)	0.37	329 (−278 to 935)	0.25
Posterior longitudinal ligament opening	−8465 (−25,948 to 9018)	0.33	−0.41	24,111 (−14,968 to 63,190)	0.20	10,890 (−35,214 to 56,993)	0.61
Disc fragment removal	−31 (−402 to 341)	0.86	0.02	159 (−295 to 614)	0.45	241 (−276 to 758)	0.32

Abbreviations: CI, confidence interval; sec, seconds. ^a^ Adjusted mean differences from mixed-effects models; negative values indicate faster performance with 3D visualization.

**Table 4:** Subjective Workload Scores by Visualization Modality and Expertise Level.

Group	Condition	Mean ± SD	Adjusted EMM (95% CI) ^a^	Within-Group Difference (2D vs. 3D), Mean (95% CI)	*p*-Value	Cohen’s d
Seniors (*n* = 3)	2D	28.4 ± 12.1	26.8 (11.8 to 41.8)	1.6 (−6.6 to 9.8)	0.67	−0.81
	3D	26.8 ± 10.5	25.2 (10.1 to 40.2)	-	-	
Residents (*n* = 3)	2D	48.9 ± 14.7	47.1 (32.1 to 62.1)	−3.5 (−12.8 to 5.8)	0.42	0.41
	3D	52.3 ± 12.8	50.6 (35.6 to 65.6)	-	-	
Overall (*n* = 6)	2D	38.7 ± 16.2	38.1 (22.4 to 53.8) ^b^	−0.9 (−5.7 to 3.9)	0.70	0.19
	3D	39.6 ± 15.9	39.0 (23.1 to 54.9) ^b^	-	-	

Abbreviations: 2D, two-dimensional visualization; 3D, three-dimensional visualization; CI, confidence interval; EMM, estimated marginal mean; SD, standard deviation. ^a^ Estimated marginal means adjusted for spinal segment, operative side, and session order. ^b^ Pooled analysis without expertise interaction term.

**Table 5:** User Experience, Ergonomic Assessments, and Image Quality Ratings by Visualization Modality.

Outcome	2D, Mean ± SD or Median [IQR]	3D, Mean ± SD or Median [IQR]	Adjusted Effect (95% CI) ^a^	*p* Value	Cohen’s d
**Continuous outcomes (0–100 scale)**					
Satisfaction with performance	61.8 ± 26.7	67.2 ± 22.7	β = 2.0 (−39.0 to 43.1)	0.92	0.14
Fatigue	21.8 ± 20.9	26.2 ± 23.0	β = 0.6 (−16.7 to 17.9)	0.94	0.54
Distraction	20.0 ± 16.2	18.3 ± 15.3	β = 7.5 (−12.1 to 27.1)	0.41	−0.23
Task complexity	39.7 ± 24.5	43.2 ± 25.3	β = 3.2 (−21.7 to 28.2)	0.78	0.37
Situational stress	18.5 ± 20.2	17.0 ± 17.5	β = 13.0 (−8.0 to 34.0)	0.17	0.56
**Ordinal outcomes (Likert scale 1–5)**					
Surgical ergonomics	4 [4–5]	4 [4–5]	OR = 0.45	0.59	
Surgical working zone	4 [4–4]	4 [4–4]	OR = 1.51	0.73	
Hand–eye coordination	2 [1–3]	2 [2–4]	— ^b^	— ^b^	
**Binary outcomes (% satisfactory/yes)**					
Depth of field	100%	91.7%	— ^c^	—	
Image resolution	100%	75.0%	— ^c^	—	
Image sharpness	100%	66.7%	— ^c^	—	
Image luminance	100%	75.0%	— ^c^	—	
Image contrast	91.7%	100%	— ^c^	—	
Monitor adjustment required	8.3%	33.3%	— ^c^	—	
Visual artifacts present	16.7%	8.3%	— ^c^	—	
Eyestrain present	0%	8.3%	— ^c^	—	

Abbreviations: CI, confidence interval; OR, odds ratio; sec, seconds. ^a^ Adjusted mean differences from mixed-effects models; negative values indicate faster performance with 3D visualization. ^b^ Effect estimate could not be reliably obtained because of sparse response distributions and insufficient variability across rating categories. ^c^ Inferential effect estimates were not calculated because of sparse data and zero or near-zero cell counts; results are presented descriptively only.

## Data Availability

All data generated or analyzed during this study are included in this published article and its Supplementary Materials. Additional details or extracted datasets are available from the corresponding author upon reasonable request.

## Supplementary Materials

The following supporting information can be downloaded at https://www.mdpi.com/article/10.3390/neurosci7050097/s1: Table S1: Consort 2025 Checklist; Table S2: Scoring Sheet.

## Abbreviations

The following abbreviations are used in this manuscript:

2D	Two-dimensional
3D	Three-dimensional
AR	Augmented reality
CI	Confidence interval
CONSORT	Consolidated Standards of Reporting Trials
EMM	Estimated marginal mean
Hz	Hertz
IQR	Interquartile range
NASA-TLX	National Aeronautics and Space Administration Task Load Index
OR	Odds ratio
SD	Standard deviation
Sec	Seconds
UBE	Unilateral biportal endoscopic
